# From digital world to real life: a robotic approach to the esophagogastric junction with a 3D printed model

**DOI:** 10.1186/s12893-019-0621-6

**Published:** 2019-10-25

**Authors:** Luigi Marano, Alessandro Ricci, Vinno Savelli, Luigi Verre, Luca Di Renzo, Elia Biccari, Giacomo Costantini, Daniele Marrelli, Franco Roviello

**Affiliations:** 1 Department of Medicine, Surgery and Neurosciences, Unit of General Surgery and Surgical Oncology, University of Siena, Strada delle Scotte, 4 - 53100 Siena, Italy; 2 3dific srl, Innovative Startup, Perugia, Italy

**Keywords:** 3D printing, Anatomy, Technology, Model, Robotic surgery, Esophageal surgery

## Abstract

**Background:**

Three-dimensional (3D) printing may represent a useful tool to provide, in surgery, a good representation of surgical scenario before surgery, particularly in complex cases. Recently, such a technology has been utilized to plan operative interventions in spinal, neuronal, and cardiac surgeries, but few data are available in the literature about their role in the upper gastrointestinal surgery. The feasibility of this technology has been described in a single case of gastroesophageal reflux disease with complex anatomy due to a markedly tortuous descending aorta.

**Methods:**

A 65-year-old Caucasian woman was referred to our Department complaining heartburn and pyrosis. A chest computed tomography evidenced a tortuous thoracic aorta and consequent compression of the esophagus between the vessel and left atrium. A “dysphagia aortica” has been diagnosed. Thus, surgical treatment of anti-reflux surgery with separation of the distal esophagus from the aorta was planned. To define the strict relationship between the esophagus and the mediastinal organs, a life-size 3D printed model of the esophagus including the proximal stomach, the thoracic aorta and diaphragmatic crus, based on the patient’s CT scan, was manufactured.

**Results:**

The robotic procedure was performed with the da Vinci Surgical System and lasted 175 min. The surgeons had navigational guidance during the procedure since they could consult the 3D electronically superimposed processed images, in a “picture-in-picture” mode, over the surgical field displayed on the monitor as well as on the robotic headset. There was no injury to the surrounding organs and, most importantly, the patient had an uncomplicated postoperative course.

**Conclusions:**

The present clinical report highlights the feasibility, utility and clinical effects of 3D printing technology for preoperative planning and intraoperative guidance in surgery, including the esophagogastric field. However, the lack of published data requires more evidence to assess the effectiveness and safety of this novel surgical-applied printing technology.

## Introduction

The importance of intraoperative safety for both patients and surgeons and the concept of “tailored surgery” have become one of the main topics in surgical research over the past few years [1]. Patient-centered preoperative planning is required to achieve accurate knowledge of the target anatomy, thereby helping surgeons during critical steps and potential complications [2]. In recent years, the rise of robot-assisted surgery for a variety of surgical procedures has significantly reshaped surgical practice [3–5], although the safety of the patient remains essential. The robotic platform enables surgeons to operate more accurately during difficult procedures compared to conventional laparoscopy, which provides high resolution three-dimensional (3D) operative views and improves depth perception, as well as superior instrument handling [6, 7]. 3D printing, in addition to the standard medical imaging, may represent an invaluable tool to allow a good representation of surgical scenario, particularly in challenging cases [8]. Additionally, the 3D models provide the surgeon with an opportunity to review, plan, and study the procedure in detail even days before the surgery.

However, from its first description in the 1980s, 3D printing has been limited in maxillofacial and orthopedic surgeries and in particular for implants and prostheses [9, 10]. Recently, the technology has been extended to spinal surgery, neurosurgery as well as cardiac surgery [11–15]. Evidence in gastrointestinal surgery is still lacking. Here we report the first case using the combination of 3D printing technology and robotic esophagogastric surgery in a condition of achalasia with complex anatomy due to a markedly tortuous descending aorta.

## Materials and methods

**Fig. 1:**
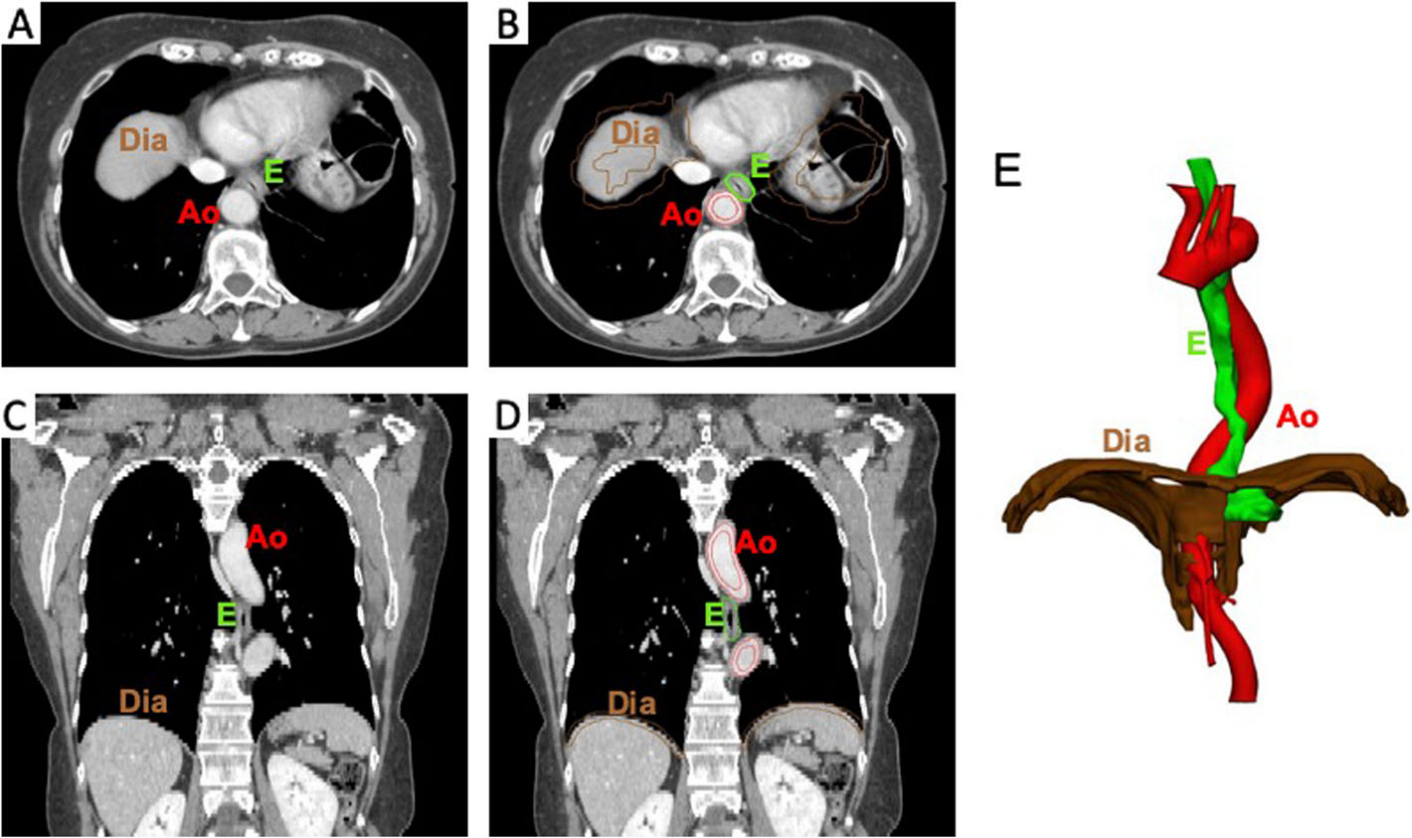
(**a**) Basal axial (transverse) image of CT scans of the thorax/upper abdomen. (**b**) Segmented axial (transverse) image of CT scans of the thorax/upper abdomen. (**c**) Basal coronal image of CT scans of the thorax/upper abdomen. (**d**) Segmented coronal image of CT scans of the thorax/upper abdomen. (**e**) Final 3D reconstruction. E, Esophagus; Ao, Aorta; Dia, Diaphragm

A 65-year-old Caucasian woman presented with heartburn and pyrosis. She also experienced intermittent mild dysphagia for solid food. The patient had no history of other relevant diseases and no abnormal family or medication history of note. She denied prior weight loss, halitosis, ingestions of caustic substances, excessive alcohol consumption, hematemesis, and melena. The physical examination was unremarkable. No abnormalities were found at conventional esophageal barium swallow and upper endoscopy revealed evidence of Barrett’s esophagus histologically confirmed as uncomplicated intestinal metaplasia. A lower esophageal sphincter (LES) hypotension, shortening of the abdominal LES, ineffective peristalsis and type 2 esophagogastric junction (EGJ) subtype (small hiatus hernia) resulted from esophageal high-resolution manometry (HRM). Additionally, a series of transmitted cardiac pulsations unrelated to swallows were found on the distal esophagus just above the EGJ. A thorax CT scan was helpful in define the anatomical relations of the esophagus with the major thoracic vessels. It highlighted a compression of the esophagus between a tortuous thoracic aorta and left atrium, allowing to diagnose a dysphagia aortica. Thus, surgical treatment of anti-reflux surgery with separation of the distal esophagus from the aorta was planned. To define the strict relationship between the esophagus and the mediastinal organs, a life-size 3D printed model of the esophagus including the proximal stomach, the thoracic aorta and diaphragmatic crus, based on the patient’s CT scan, was manufactured. The imaging data were segmented to outline the relevant structures then converted to a 3D triangulated surface mesh file suitable for fabrication with the assistance of a 3D printing and manufacturing company (3dific Srl, Perugia, Italy) (Fig. 1).

**Fig. 2:**
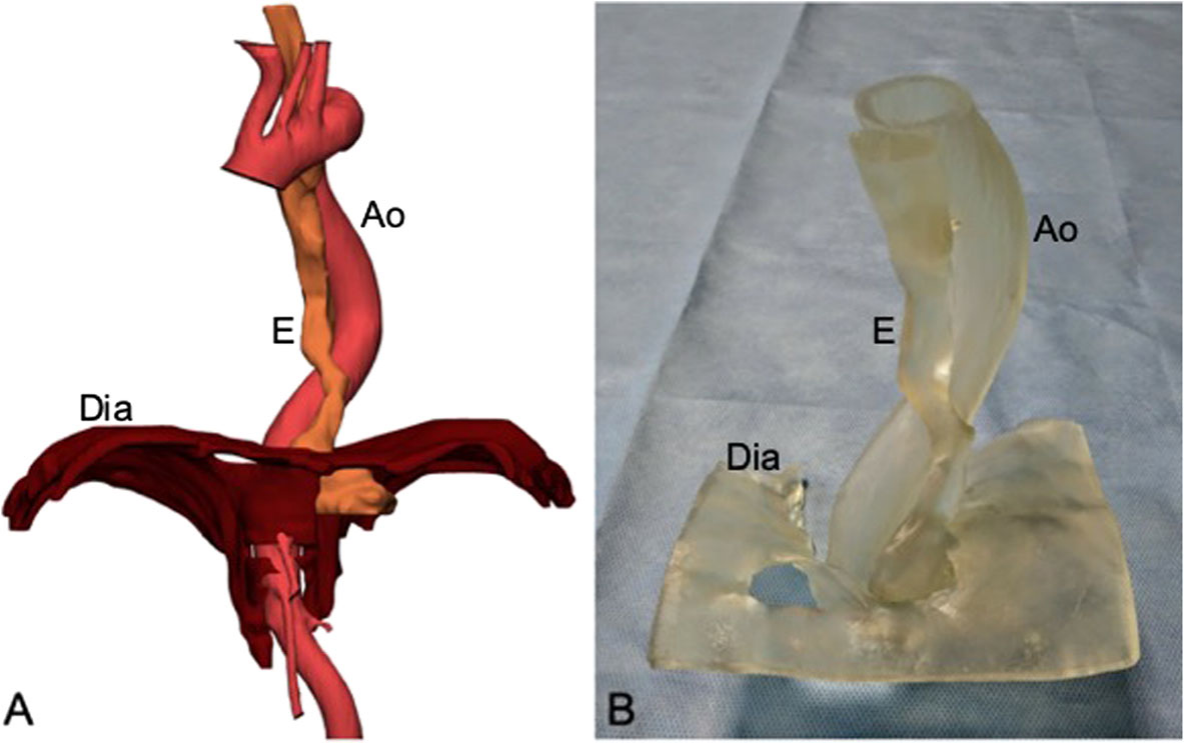
(**a**) The imaging data were segmented to highlight and color code the distal esophagus, upper third of stomach, aorta and diaphragmatic crus. (**b**) A virtual model was converted into 3D printed model, made of photopolymer resin, showing distal esophagus and its relationship with the mediastinal structures. E, Esophagus; Ao, Aorta; Dia, Diaphragm

The segmentation and design of model were reviewed for accuracy by the surgeon and engineer. Finally, the model was realized in 48 working hours by means of a stereolithography 3D printer selectively curing resin (Fig. 2). The cost associated with 3D printed model production was 230,00 Euros. Using the 3D model of the esophago-gastric junction allowed surgeon to preoperatively locate the general position and proximity of tortuous thoracic aorta with the esophagus as well as surrounding tissues. Particularly, the surgical team measured the thoracic aorta positioning and esophago-gastric structures on the 3D model before operating then applied the patient-tailored surgical anatomy in the surgical field. This model enabled surgeons to verify the position of critical structures and to discuss all possible approaches and strategies to operate as well as plan all possible critical maneuvers. This provides proof that the operation can be performed safely through less invasive techniques, without using conventional approach to dominate such complex case. Institutional review board approval was not required, since the privacy and personal identity information of patient were protected, i.e., all the data were analyzed anonymously, and the patient was treated with approved diagnostic and therapeutic procedures according to generally accepted standards of care. Anyway, the patient gave informed written consent to participate to this study.

## Results

**Fig. 3:**
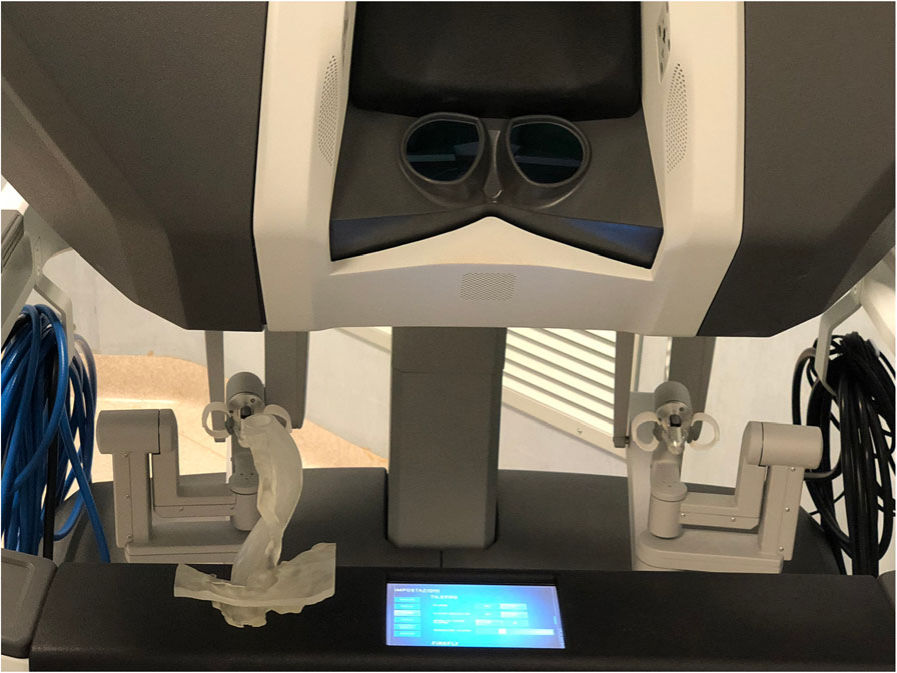
Model in the operating theatre with the operating robot ready to use

**Fig. 4:**
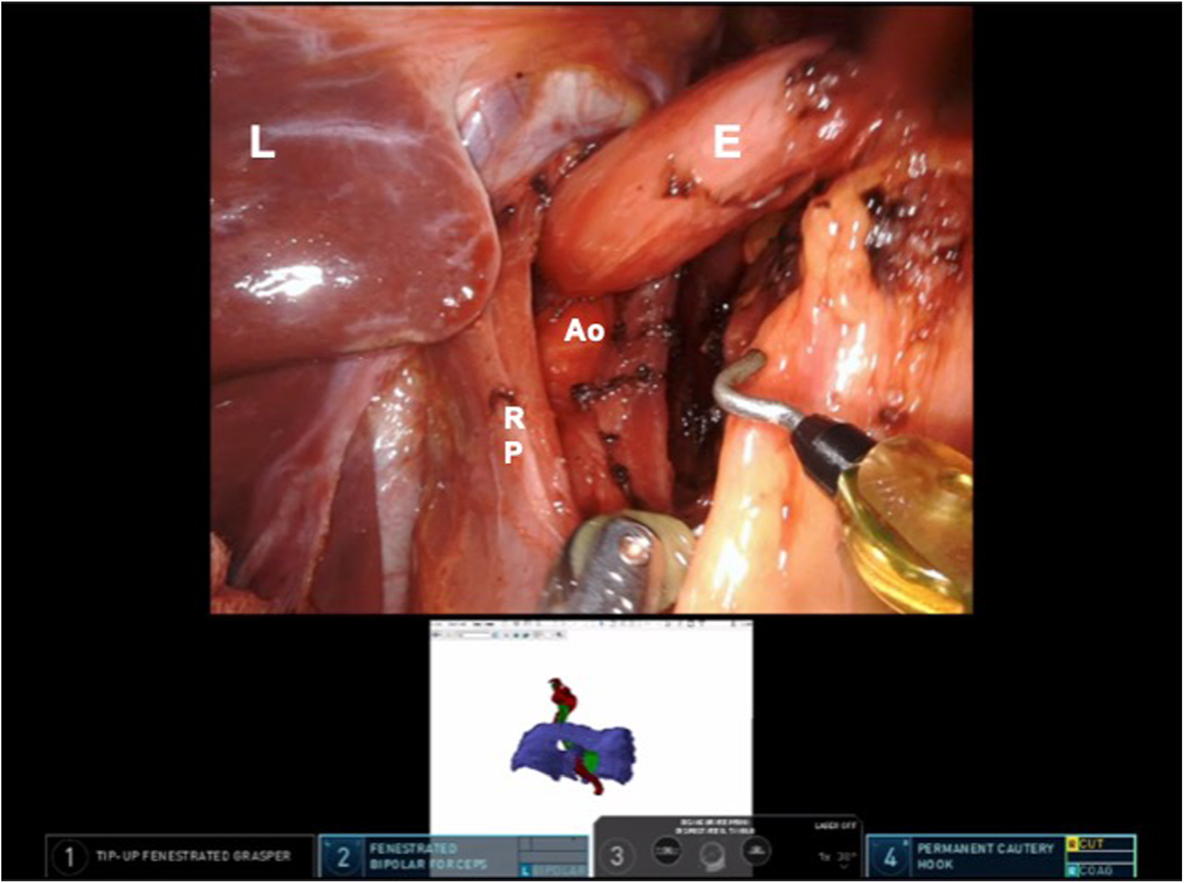
Intraoperative view of the patient anatomy highlighting the “picture-in-picture” mode. E, Esophagus; Ao, Aorta; RP, Right Pillar; L, liver

The robotic procedure was performed with the da Vinci Surgical System (Xi, Intuitive Surgical Inc., Sunnyvale, CA, USA). The manipulation of 3D prototype each time deemed necessary by on-console surgeon ensured adequate orientation on the surgical field and identification of critical anatomic landmarks (Fig. 3). Furthermore, the surgeons had navigational guidance during the procedure, since they could consult the 3D processed images electronically superimposed, in a “picture-in-picture” mode, over the surgical field displayed on the monitor system and on a robotic headset. The procedure started with the division of gastro-hepatic ligament and dissection of the phreno-esophageal membrane to expose and dissect the diaphragmatic pillars. A retro-esophageal window was obtained for the positioning of an umbilical tape, pulling up the esophagus and exposing the hiatus. Periesophageal mediastinal dissection was initiated bluntly, taking care to preserve the aortic plane behind the esophagus. This step was carried out very carefully and thermal devices were limited during dissection to prevent any vascular injuries. Furthermore, the separation of the esophagus from the aorta was obtained as far as possible into mediastinum until 3 cm of the esophagus were transposed into the abdomen under no tension (Fig. 4). The crura were then closed from the right of the esophagus with interrupted non-absorbable sutures placed 8 to 10 mm apart, 10 mm back from the crural edge. A Nissen fundoplication was then completed. There was no injury to the surrounding organs. The total operative time was 175 min. The patient had an uncomplicated postoperative course and was discharged home on the third postoperative day.

## Discussion

In this case report, we assessed the feasibility, utility and clinical impact of a novel surgical-applied technology for preoperative planning and intraoperative guidance for a rare case of dysphagia aortica treated by robotic approach. Although three-dimensional printing was described for the first time more than three decades ago, its diffusion in digestive surgery has increased only over the last years [16]. Nevertheless, few data are still available in the literature about its role in esophageal surgery. Dickinson KJ et al. [11] described for the first time the application of 3D modelling to complex esophageal cases. In a patient after a left pneumonectomy and thoracic aorta replacement by means of Dacron graft, complicated by an aorto-esophageal fistula requiring aortic bypass and esophageal diversion with feeding gastrostomy, three-dimensional modelling facilitated the complex definitive endoscopic-surgical treatment. The endoscopic mucosal resection, as well as argon plasma ablation of excluded esophagus followed by transection of the distal esophagus, was allowed by the preoperative treatment simulation and intraoperative model manipulation to obtain an accurate real-time recognition of critical anatomic landmarks. In the same way, another 3D model was used in a patient with multiple esophageal diverticula facilitating the most appropriate surgical strategy in a preoperative setting. To the best of our knowledge, no other study addressing the application of the 3D printed model in upper gastrointestinal surgery is conducted and no consistent data as regard its usefulness are available. Here we demonstrated the feasibility of creating a rigid life-size 3D printed model of the esophagus including the thoracic aorta, the upper third of the stomach, and diaphragmatic crus, using images imported from the standard computerized tomography for the purpose of preoperative procedure planning and external guidance in the intraoperative setting. The preoperative study of the patient-tailored surgical anatomy represents one of the cornerstones of 3D printing. Hamabe et al. [17] created a 3D printed pelvic model to improve the comprehension of pelvic anatomy during laparoscopic surgery for rectal cancer. As a result, the preliminary understanding of complex anatomical relations was reflected in a more safe and effective intervention. In the same way, Garcia-Granero A et al. [18] proposed a 3D model for preoperative planning of superior mesenteric as well as ileocolic vascular pattern to facilitate the lymphadenectomy during complete mesocolic excision for right colon cancer. They discussed that the walk down of vascular roots before laparoscopic right hemicolectomy could reduce the risk of venous injury and intra-operative hemorrhage. The advantage of technological development to create 3D model and tool for surgical use from CT image elaboration is represented by the recognition, in a real size mode, of the interspatial relationship between anatomical structures of the target area [19]. Furthermore, the availability of the model several days prior to surgery represents the mainstay of 3D printing technology: all surgical team members can discuss the surgical strategy evaluating all possible approaches and solutions to perform the operation as well as defining critical maneuvers in a calmer condition than surgical theatre. In the reported case, the 3D model improved the preoperative discussions with better evaluation of the target anatomy and helped the surgeon to decisions to proceed with robotic rather than laparoscopic, open transabdominal as well as transthoracic approach. During the operation, the visualization of the tortuous thoracic aorta and its proximity to the distal esophagus from the 3D model was fundamental to the safe outcome of the procedure, allowing the surgeons to recognize in detail the relative positions of critical structures. In this way, a real model in the surgeon’s hands overcame the absence of tactile feedback of the robotic technology and the limitations of 3D CT images manipulation on the screen. Reducing operative time represents another important advantage [20]. The preoperative team discussion and the preliminary intervention plan with the evaluation of all possible solutions, the definition of dissection planes, and the simulation of critical maneuvers allowed the surgeon to focus on other key points resulting in a safer surgery [20, 21]. Moreover, it is important to emphasize that the 3D printed model influences the planning of the kind of surgery. Specifically, in the absence of preoperative recognition of critical structures as well as interspatial relationship between anatomical structures of the target area by means of 3D model, the operation would not have been conducted with the robotic approach. Accordingly, in recent years have been reported encouraging results of pre-surgical simulation on a patient-specific tissue-like 3D model [22]. The recent technological advances lead to print any kind of human parts, which can be made of soft and deformable materials mimicking the physical properties of human tissues [8, 19, 23]. Surgeons of any disciplines and expertise have the chance to improve their surgical skills through multiple repetitions of the same maneuvers outside the operating room getting ready for a real intervention before it is carried out on the patient. Von Rundstedt et al. [22] described their experience on 10 patients with renal tumors who underwent robot-assisted laparoscopic partial nephrectomy after preoperative rehearsal using 3D silicone patient-specific renal models. They demonstrated the same results in tumor resection time, resected tumor volume and morphology between the model and patient’s kidney, suggesting that the simulation platform may represent an invaluable tool for surgical decision-making, preoperative rehearsal as well as surgical training. Similar results were reported also by Pugliese L et al. [19] after preoperative simulation of robotic live-donor nephrectomy and robotic correction of splenic artery aneurysm. They highlighted that the training on the same patient’s anatomy could increase the confidence during the operation. Of course, this would be one of the major advantages over conventional as well as a virtual reality-based surgical simulator for minimally invasive technique. Moser A et al. [24] conducted a studied aiming at underlyine the difference in experience between digital and physical handling in brick-puzzle games. Interestingly, they report that resistance conveys a kind of inertia by means of the fingers feeling the tactile sense, in the experiment conducted in a real world. In the virtual reality, on the contrary, this advantage is lost even if the software included a feedback mechanism. They postulated that the computer may not produce the same detailed awareness that is created via our many sensory experiences. Virtual feedback will therefore always be unsatisfactory because it is not anchored in the real life [25].

The main limitations of 3D printing included the time as well as relatively high costs of production.

The printing process, from the elaboration of the image to production of the model, could take hours to several days [21, 26, 27]. Of course, the more complex the model, the more time is needed to its production. Actually, for these reasons, the 3D printing remains a prerogative of elective surgery [26, 27]. It is of note that, since its primary use consists of preoperative planning, training, and simulation, 3D printing should be prepared in advance in order to be properly applied. It is claimed that variable costs depend on the type of printing technique, materials used, and workload of dedicated staff [20, 21, 26, 27]. Anyway, the sharing of the printing platform and the optimization of production staff members by multiple users could reduce the expenditures [26].

## Conclusions

The technological innovations, such as 3D printing and robotic surgery, represent an unimaginable progress until a few years ago. The present report underlines the feasibility, advantage and clinical impact of 3D printing technology for preoperative planning and intraoperative guidance for esophagogastric surgery. However, the lack of published data requires more evidence to assess the effectiveness and safety of this novel surgical-applied printing technology.

## Data Availability

Data sharing is not applicable to this article as no datasets were generated or analyzed during the current study.

## Abbreviations

3D: three-dimensional
EGJ: esophagogastric junction
HRM: high resolution manometry
LES: lower esophageal sphincter
